# Development and Validation of a Spike Detection and Classification Algorithm Aimed at Implementation on Hardware Devices

**DOI:** 10.1155/2010/659050

**Published:** 2010-03-14

**Authors:** E. Biffi, D. Ghezzi, A. Pedrocchi, G. Ferrigno

**Affiliations:** ^1^Neuroengineering and Medical Robotics Laboratory, Bioengineering Department, Politecnico di Milano, Piazza Leonardo da Vinci 32, 20133 Milano, Italy; ^2^Neuroscience and Brain Technologies Department, Italian Institute of Technology, via Morego 30, 16163 Genova, Italy

## Abstract

Neurons cultured in vitro on MicroElectrode Array (MEA) devices connect to each other, forming a network. To study electrophysiological activity and long term plasticity effects, long period recording and spike sorter methods are needed. Therefore, on-line and real time analysis, optimization of memory use and data transmission rate improvement become necessary. We developed an algorithm for amplitude-threshold spikes detection, whose performances were verified with (a) statistical analysis on both simulated and real signal and (b) Big O Notation. Moreover, we developed a PCA-hierarchical classifier, evaluated on simulated and real signal. Finally we proposed a spike detection hardware design on FPGA, whose feasibility was verified in terms of CLBs number, memory occupation and temporal requirements; once realized, it will be able to execute on-line detection and real time waveform analysis, reducing data storage problems.

## 1. Introduction

Neuronal cells communicate by means of electric pulses, called Action Potentials (APs) or, briefly, spikes [1, 2]. These voltage changes have been traditionally recorded with conventional electrodes (e.g., glass pipettes), therefore the number of neurons simultaneously recorded and the time needed for electrodes placement are well known limits [3].

To overcome these experimental difficulties, the use of MicroElectrode Array biochips (MEAs) guarantees the possibility to record extracellular activity of neuronal preparations from tens of electrodes at the same time [4]. Because of the inherent nature of the extracellular recording, each electrode records the neuronal activity from a region, where generally tens of neurons are present thus providing the acquisition of a Multi Unit Activity (MUA). To extract the activity of every single firing unit influencing that electrode from the MUA, we need a process called “spike sorting” which includes AP detection and classification.

There are two different ways to acquire and analyze electrophysiological data: store the raw trace observed on all electrodes and perform spike detecting and sorting later (offline sorting) or detect and sort spikes immediately (during acquisition) and only store the sorted spikes (real-time online sorting) [5]. A compromise between these approaches is to detect spikes online and only store the detected spikes for later offline sorting.

Spike detection and classification of neuronal action potentials can be performed using supervised methods [6, 7] or using unsupervised ones [8, 9]. As the particular knowledge of the APs is not available before we detect them, supervised detectors (e.g., artificial neural networks) have obvious limits in real applications. Unsupervised methods, instead, are generally used because they do not require a priori knowledge about neuronal spike waveforms nor the user presence: thus, they are very useful for automatic processing purposes.

The most widely used unsupervised technique to detect APs is the amplitude threshold crossing. Conventional spike detection is made by comparing signal amplitude with a fixed or an adaptive threshold, depending on the Root Mean Square (RMS) value of data in running windows [10, 11]. Problems related to the large number of electrodes make automatic threshold level adjustment attractive compared to manually setting the threshold for each channel [9]. Both methods suffer from bias induced by high amplitude and high firing-rate neuronal spikes [8]; because of the silencing of biological activity is unfeasible, levels must be estimated from the recorded superposition of signal and noise. Several methods were recently proposed to overcome this difficulty by adopting an unbiased estimate of the standard deviation (SD) of background noise. Donoho [12] claimed that the median absolute deviation divided by the 75th percentile of the standard normal distribution is equal to the S.D. of background noises. Wagenaar et al. in 2005 [11] proposed a tool using different algorithms to estimate noise level. In 2007, Thakur et al. [3] developed an algorithm to estimate the SD of background noises by considering only the cap, which is the middle portion of the amplitude distribution, that is, “cap-fitting” algorithm. Moreover, researchers realized systems or chip embedded to detect the presence of neuronal spikes and communicate only active portions of recorded signals [4]. Even if these methods suffer from some problems [3] they are often used to optimize detection efficiency. Methods such as principal components [13] and Haar transform [14] have also been used. Other methods involve discrimination based on different properties of AP waveforms, such as zero crossing and spike width [15] and discrimination based on conduction velocity [16]. Discrimination methods are based on forming a feature space defined by the spikes and isolating spikes by creating discrimination boundaries between clusters within that feature space. 

The methods typically used in supervised classifications for the classification of the detected action potentials, are template matching and the multilayer perceptron detectors [7, 17]. Other approaches that have been used are artificial neural networks [7] in which a training procedure is used to determine the weights of a feed-forward discrimination network to perform the classification. All these methods require prior information about the APs; for this reason unsupervised classifiers are preferred. Among the unsupervised spike sorting systems, the Principal Component Analysis (PCA) [6] and the fuzzy c-means (FCM) clustering [18] are commonly used. Classifier methods have been compared by Wheeler and Heetderks [19], evaluating the performances of nine different methods including spike amplitude, conduction latency, PCA and template matching using Euclidean distance. The last two methods were found to perform the best for spike sorting in noisy data. In this approach, templates that represent a typical waveform are used as a standard. A dissimilarity measure is then defined and the unknown input spikes are compared with the standard. Several dissimilarity measures have been used [3]. A new clustering technique is the hierarchical classifier. In this approach, clusters are successively grouped into greater ones. 

Our goal meets the increasing demand for long period recordings to study the functional and adaptive properties of complex neuronal networks on which plasticity and learning are based. 

The present study deals with the selection and validation of software for the adaptive detection and classification of neuronal spikes, able to distinguish the activity of single cells from the signals recorded by MEA. We have to meet two important requirements: operate in real-time and work for long periods. Thus, spike detection must be adaptive, unsupervised and fast. Hence, this task could clearly benefit from the acceleration achievable with FPGAs (Field Programmable Gate Arrays). In 2005, Zviagintsev et al. [20] proposed 3 VLSI (Very Large Scale Integration) architectures for processing of data coming from an implantable integrated circuit; their architectures were power-efficient but must be implemented for each channel. To assure fast processing for online detection on 60 electrodes, our algorithm will be integrated on an FPGA. Therefore, it needs to be simple for the hardware implementation on VHDL language (VLSI Hardware Description Language). Spike detection will be nonreversible, since only spike templates are saved, as to reduce data storage. Moreover, to obtain an on-line classification, waveforms extracted will be sent to a Digital Signal Processor (DSP) for automatic classification.

## 2. Materials and Methods

### 2.1. Neurons Cultures

Hippocampal neurons from new-born (P0) mice pups were prepared following [21]; cells were mechanically dissociated using trituration after a treatment with trypsin at 37°C. Cells were then plated at 40,000 cells/chip on MEAs treated with poly-L-lysine (0.1 mg/ml). MEA60 (Ayanda Biosystems SA, Lausanne, Switzerland) with platinum (Pt) 40 *μ*m round electrodes, 200 *μ*m spaced were utilized. Cells were cultured in Neurobasal supplemented with B27 (2%), Pen/Strep (1%) and L-glutamine (2 mM). 50% medium was changed every week. 

Then, we recorded spontaneous activities of cultured hippocampal neuronal network on MEA substrate at 21 Days in Vitro (DIV), about the complete maturation of the network. Extracellular recordings were carried out with a MEA1060 signal amplification and data acquisitions system (Multi Channel Systems MCS GmbH, Reutlingen, Germany). Sampling rate was 25 000 Hz and single recordings lasted 300 s.

### 2.2. Algorithms Design

**Figure 1:**
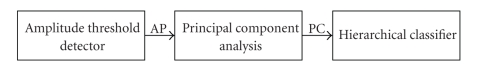
Block diagram of the algorithm design.

The algorithm, developed in Matlab (The Mathworks, Natick, USA) environment, is made up of three focal elements. First of all, an amplitude-threshold spikes detector, based on noise level estimation, identifies peaks over threshold, APs. Then APs are bundled into group using PCA. Finally, they are classified with a hierarchical classifier (Figure 1).

The complete algorithm was tested first on simulated data and then on neuronal spikes, which were obtained recording spontaneous activities from hippocampal neuronal networks cultured on MEA substrates.

**Figure 2:**
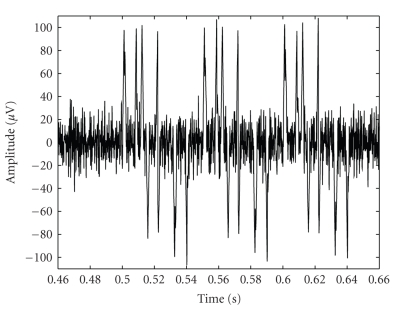
Simulated signal (200 ms).

The 60 seconds simulated signal (Figure 2) was artificially made up of both positive and negative triangular waves (each wave is composed by 20 samples); the intensity peaks range from 40 to 100 *μ*m. In addition, 2 overlapped and temporally shifted triangular waves, representing multi-shaped AP in neuronal culture activity, were included in the simulation. The full amount of valid spikes, simple and complex waves, was 600.

A white Gaussian noise was used to simulate background noise. First, it was band-pass filtered (2nd order Butterworth) between 150 Hz and 2,500 Hz, then normalized and overlapped to simulated signal. The Signal to Noise Ratio (SNR) is equal to 5 dB, as real neuronal culture SNR [22].

#### 2.2.1. Spikes Detection

The spikes detection algorithm is structured as follows. First, the signal is band-pass filtered with a 2nd order Butterworth filter (150 Hz–2,500 Hz); then, both positive and negative thresholds values are determined as a multiple of the noise level esteem. Finally, positive APs over positive threshold and negative APs under negative threshold are detected and validated to prevent double detections of unitary events. A detected peak is tested in ±1 ms window, checking if it is the highest peak of either polarity and if the 50% of its amplitude is higher than other peak of the same polarity, as discussed by Wagenaar et al. [11]. If both tests are passed, the detected peak is consider an AP; otherwise it is rejected. A specific activity was dedicated to the definition of thresholds as well as to the selection of the noise level esteem.

Five noise level estimation algorithms, some suggested by literature [11, 23], some modified were developed. Then, their performances were compared with statistical analysis, to select the best solution.

(a) *BandFlt.* Based on the algorithm described in [11, 23], detects spikes if the filtered stream exceeds a given multiple (supposed equal to 4) of the estimated RMS noise. Three hundred 10 ms windows of data are read and for each of these windows the RMS values are computed. The results are sorted, and the final esteem of noise level is the 25th percentile of the values collected. Positive and negative threshold values are equal and fixed.
(b) *Limada* [11, 23]. Like BandFlt, it splits the data stream into 10 ms windows, and determines the 2nd (*V*_.02_) and 30th (*V*_.30_) percentiles of the distribution of voltages found in each window. Then, it performs two tests: it checks if the ratio between *V*_.02_ and *V*_.30_ is smaller than 5 (i.e.: no actual spike in the window) and if the absolute value of *V*_.30_ is significantly non-zero (i.e.: data in the window are not blanked out). If both tests are passed, the window is considered “clean”. Noise level initialization value is defined after the collection of 100 clean windows; then the current noise level estimate (noise_est) is updated as follows:
  (1) $$\text{noise}\_\text{est}\left(k\right)=0.99\times \text{noise}\_\text{est}\left(k-1\right)+0.01\times \left|V_{.02}\right|,$$
  where *k* is the current iteration index.

Positive and negative threshold values are computed by multiplying noise level estimate by 4. Their values are opposite and adaptive.

(c) Another adaptive algorithm suggested by literature [11, 23] and tested in this work is *AdaFlt*. It divides the signal into 10 ms windows and 128 windows of data are read. For each of these windows, the maximum and minimum values are measured; then results are sorted and 40th percentile of both collections is computed (*M*_.4_ and *m*_.4_). The noise level initialization estimates for positive (noise_est*_p_*) and negative (noise_est*_n_*) spikes are based on these data. Thresholds are computed by multiplying noise level esteem by 2. While running, AdaFlt keeps collecting minima and maxima in 10 ms windows, although it uses only one every ten windows. Whenever 128 windows have been collected, positive (2) and negative (3) thresholds are updated as

(2) $$\begin{array}{c} \begin{array}{cc} & \text{noise}\_\text{es}{\text{t}}_{p}\left(k\right)=0.9\times \text{noise}\_\text{es}{\text{t}}_{p}\left(k-1\right)+0.1\times M_{.4}, \end{array} \end{array}$$

(3) $$\begin{array}{c} \begin{array}{cc} & \text{noise}\_\text{es}{\text{t}}_{n}\left(k\right)=0.9\times \text{noise}\_\text{es}{\text{t}}_{n}\left(k-1\right)+0.1\times m_{.4}. \end{array} \end{array}$$

(d) *AdaFlt 128* differs from AdaFlt because AdaFlt 128 adapts thresholds after 128 windows, AdaFlt after 1280. Therefore AdaFlt 128 is faster and more adaptive than AdaFlt, but it suffers more for noise fluctuations.
(e) The last algorithm developed in this work is *AdaBandFlt*, devised as an improvement of BandFlt with adaptive properties. It splits the data stream into 10 ms windows, calculates the RMS values for each of the initial 100 windows and determines the 25th percentile of RMS distribution (*N*_.25_). Noise level initialization value is defined. Then the current noise level esteem is updated as follows:
  (4) $$\text{noise}\_\text{est}\left(k\right)=0.8\times \text{noise}\_\text{est}\left(k-1\right)+0.2\times N_{.25.}$$

#### 2.2.2. Spikes Classification

After detecting temporal occurrences of APs, validated waveforms must be extracted from data stream applying a 2 ms symmetric window to the signal. Then their tallest peaks (i.e., time stamps) are aligned, making up a matrix *N* × *c*, where *N* is the number of “observation” (i.e., neuronal waveforms) and *c* is the number of sample, that is, 50 for the 2 ms window. The strong assumption is that on each electrode neurons usually generate spikes with a characteristic shape; so the aim of classification is correlating source with a characteristic waveform in the recorded data. This assumption implies that neurons maintain the shape of the AP and that the whole system does not move or change.

The classification algorithm proposed in this work is based on PCA and hierarchical classification. 

The PCA finds an ordered set of orthogonal basis vectors that capture the directions of the largest variation in the data [6]. APs are bounded into groups, projecting data set onto the principal components. 

The hierarchical classifier is a method of cluster analysis; it splits *N* observations into a series of *M* clusters, where *M* can range from 1 (all observations grouped into one cluster) to *N* (each observation is a cluster). The strength of this technique is that it provides the possibility of increasing or decreasing the number of clusters depending on the required level of aggregation [24].

The hierarchical classification algorithm theoretically starts with *N* clusters, each containing just one object. Then the likeness between each couple of observation is evaluated computing the Euclidean distance between all combinations of the centroids of the clusters. Data are combined in neighbours couples, making up a binary tree diagram. Then, these binary clusters are grouped forming bigger clusters until a hierarchical tree diagram is obtained.

After the clustering, the number of spikes in each cluster is determined; 10 was defined the minimum cluster dimension not to be rejected [3]. Moreover, for each cluster the data dispersion (*D_i_*) and the centre of mass (Cm*_i_*) are computed. The hierarchical level, significant for the grouping, was defined when the centres of mass distances (Cm*_i-j_*) between one cluster (cluster*_i_*) and the others (cluster*_j_*) was greater than *D_i_*, as in

(5) $$\begin{array}{cc} D_{i}<\text{C}{\text{m}}_{i}-\text{C}{\text{m}}_{j} & \;\forall i,j=1,\ldots ,\text{no}\;\text{of}\;\text{clusters} \end{array}.$$

In literature, generally, clusters must house more than one element. However, we have decided not to constrain the number of elements in each cluster because it's possible that the best data grouping is characterised by the presence of one cluster with only one element detected, having one neuron spiking very rarely. Thus, we prefer to eventually exclude this cluster at the end of classification, avoiding inaccurate possible re-arrangements which are often mistaken.

### 2.3. Evaluation of Performance

Performances of spike detection algorithms were evaluated with statistical screening tests and, subsequently, with the big O notation.

#### 2.3.1. Algorithms Screening Tests

Algorithms performances were statistically evaluated using both the simulated neuronal signal described in Section 2.2 and the neuronal spikes identified in 5 s MEA recordings by experts' visual inspection.

They were weighed up performing a screening test (Algorithms Screening Tests) that determines True and False Positive (TP-FP) that are, respectively, APs and noise peaks detected by the algorithm considered; moreover, screening test determines True and False Negative (TN-FN) values that are, respectively, noise peaks and APs not identified. Using these 4 parameters we computed Sensibility (Se), the probability that a noise peak could be detected by algorithm, and Specificity (Sp), the probability that an AP could not be detected by algorithm. Moreover, we evaluated Positive Predicted Value (PPV), the probability that a test-positive peak should be an AP, and Negative Predicted Value (NPV), the probability that a test-negative peak should be noise. The parameters (6) are mathematically described as

(6) $$\begin{array}{c} \text{Se}=\frac{\text{TP}}{\text{TP}+\text{FN}},\\ \text{Sp}=\frac{\text{TN}}{\text{TN}+\text{FP}},\\ \text{PPV}=\frac{\text{TP}}{\text{TP}+\text{FP}},\\ \text{NPV}=\frac{\text{TN}}{\text{TN}+\text{FN}}. \end{array}$$

This evaluation guided us to identify 2 algorithms with good performances; to choose between them, the big O Notation was used.

#### 2.3.2. Big O Notation

Big O notation allows to quantitatively evaluate the performance or complexity of an algorithm as a function of the number of its input data. It describes the worst-case scenario, and can be used to illustrate the execution time required or the space used (e.g., in memory or on disk) by an algorithm. This allows designers to determine which of multiple algorithms to use, in a way that is independent of computer architectureclock rate.

The Big O Notation is defined as O(f(*N*)), with *N* the number of input data. 

We assessed the two best algorithms identified with this complexity evaluation method. To evaluate nested instructions, we considered the product between the Big O Notation of each instruction; while to assess sequentially commands, we considered only the worst and the most restraining notation.

#### 2.3.3. Classification Performances

Hierarchical classification algorithm performances were previously evaluated on simulated signal, knowing the different kinds of waveform artificially designed. On the other hand, the assessment of classification algorithm performances on real signal required visual inspection of the neuronal activity (5-second signal), formed by more AP waveforms, morphologically different. The capability of distinguishing these dissimilarities was judged comparing the visual inspection results to algorithm's output.

### 2.4. Hardware Architecture

**Figure 3:**
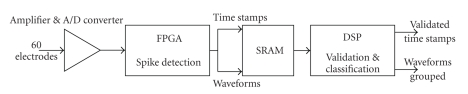
Hardware design.

The hardware design, not yet implemented, is shown in Figure 3. The input is filtered, amplified, digitalized and transferred into an FPGA, where spikes are detected. The time stamps, not yet validated, and the waveforms, are memorized in a SRAM; then they enter the DSP for the spikes validation, waveforms alignment and classification. 

FPGAs are digital circuits widely used for manufacturing complex digital systems due to the advantages they offer, such as gate count, speed, rapid hardware realization and low development cost [25]. The chip consists of a regular symmetrical structure of Configurable Logic Blocks (CLBs), interconnected by a programmable network and enhanced with lots of Input/Output Blocks (IOBs) and block RAM. Specifically, CLBs constitute the main logic resources for implementing synchronous as well as combinatorial circuits. Each CLB contains four slices and each slice contains two small Look-Up Tables (LUTs) and two flip-flops. The LUTs can be used as a 16 bits shift register or as a 16×1 distributed memory. For application requiring large, on-chip memory, block RAM is preferable than distributed RAM; it is organized in columns and each column contains a number of blocks depending on the size of FPGA [26].

The feasibility of spike detection algorithm implementation on FPGA was verified evaluating the required hardware space, in terms of CLBs number, memory occupation and timing performances. On the other hand, we decided to develop PCA on DSP, a microprocessor optimized to perform recursive instructions, as in literature [27]. Moreover, Morizet et al. [28] had already verified that the FPGA core was too slow for this computation. Finally, our design planned to develop hierarchical classifier on DSP, as already done in [29].

## 3. Results

### 3.1. Algorithms Screening Tests 

#### 3.1.1. Spikes Detection on Simulated Signal

**Figure 4:**
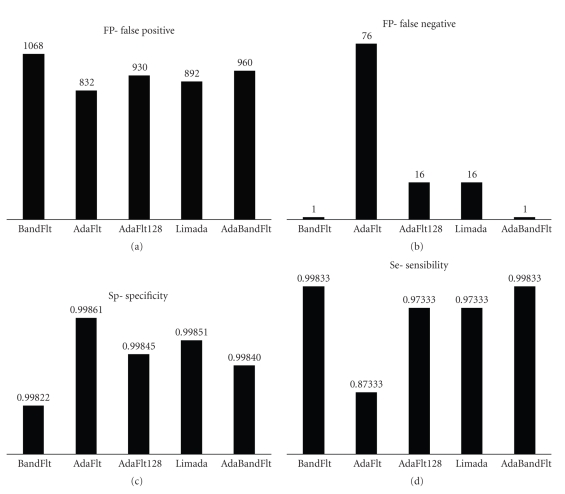
Screening test results for all the noise level estimation methods on simulated signals. (a) FP-False Positive, (b) FN-False Negative, (c) Sp-Specificity, and (d) Se-Sensibility.

Algorithms Screening Tests were performed on spikes detected but not validated in simulated neuronal signal, computing TP, FP (Figure 4(a)), TN and FN (Figure 4(b)) values.

BandFlt underestimates noise level; therefore it identifies more FPs than the others. On the other hand it produces noise level values more stable than standard deviation, confirming previous results [11, 23] and identifying only 1 FN.

AdaFlt finds the smallest number of FPs but a lot of FNs, because of the excessively slow threshold adaptation. Further, AdaFlt 128 is faster and more adaptive than AdaFlt: it identifies a small number of FNs caused by noise fluctuations.

Limada is quite reliable in order to identify the threshold: it finds a small number both of FPs and FNs. It is an adaptive method, necessary for real time analysis. The main disadvantage is that it needs approximately 5 s to initialize threshold value losing a large number of spikes.

Finally, AdaBandFlt method is like BandFlt in term of FN (i.e., 1 FN) but it finds less FP than the latter and it is an adaptive method.

Therefore we can already identify two groups of algorithms: a group characterised by finding most of FP and really few FN and a group characterised by finding very few FP and some FN. 

**Figure 5:**
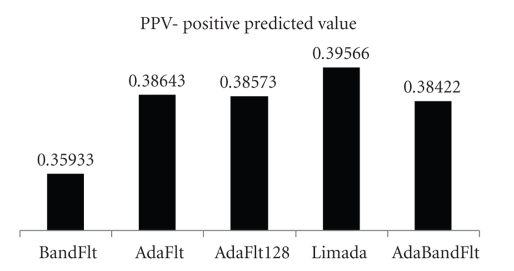
PPV-positive predicted values: evaluation between algorithms.

Then, Se (Figure 4(c)) and Sp (Figure 4(d)) were computed by means of Screening Test results. PPV was evaluated, too (see Figure 5), whereas NPV was not reported because of its low meaningfulness.

BandFlt and AdaFlt were immediately rejected because of their low value, respectively of Sp and Se.

Limada was selected because it has high Se and Sp; moreover it has the highest PPV. Likewise, AdaBandFlt was chosen because, even if its SP and PPV are comparable to AdaFlt 128, its Se is the highest one. As a matter of fact, we favoured the Se parameter preferring to reject some spike later (after classification) instead of loose true spikes at the beginning of the analysis, in view of the hardware implementation, where spike detection is not reversible.

#### 3.1.2. Spikes Detection on Neuronal Spikes

**Figure 6:**
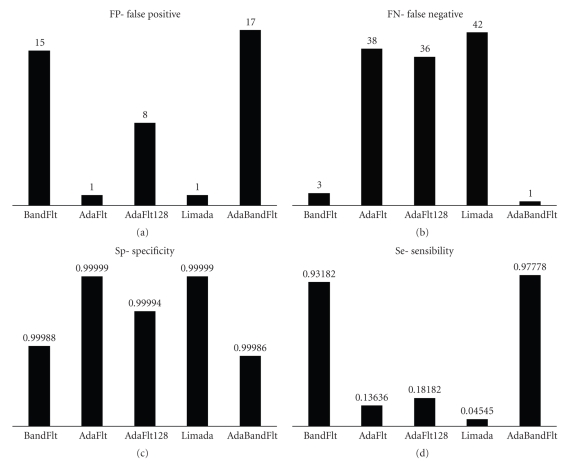
Screening test results for all the noise level estimation methods on neuronal spikes. (a) FP-False Positive, (b) FN-False Negative, (c) Sp-Specificity, and (d) Se-Sensibility.

Algorithms Screening Tests were performed on spikes detected but not validated in a 5 s fragment of a 300 s neuronal activity, recorded by MEA. TP, FP (Figure 6(a)), TN and FN (Figure 6(b)) values were computed. Then, Sp (Figure 6(c)) and Se (Figure 6(d)) were evaluated for each algorithm.

The performance evaluation of spike detection algorithms on real recorded electrical activity agreed with the analysis on simulated signal. BandFlt and AdaBandFlt find more FP than other algorithms; on the other hand AdaFlt, AdaFlt 128 and Limada miss more APs. Furthermore, differences in Se value become more evident examining neuronal activity. The performances assessment on experimental data highlights that AdaFlt, AdaFlt 128 and Limada has an extremely low sensibility on them. Differently from what was observed on simulated data, BandFlt specificity is equivalent to AdaBandFlt on experimental data. However, since the only difference between these 2 algorithms is in the adaptability, we still select AdaBandFlt, being adaptation a crucial requisite for the envisaged long term acquisitions.

### 3.2. Big O Notation

As an additional estimation, we evaluated the two best algorithms' behaviours (i.e., Limada and AdaBandFlt), regardless of the hardware, using the Big O Notation. Especially, we considered only the complexity evaluation of the threshold initialization, that was recognized as the most time consuming step of our algorithms.

The two algorithms are reported in pseudocode, to have a compact and informal high-level description; the O(f(*N*)) notation, with *N* the number of inputs, is noted in bold near each statement (see Algorithm 1).

AdaBandFlt and Limada complexity is described, respectively, by (7):

(7) $$\begin{array}{c} \text{O}\left(100^{2}\text{F}\right),\\ \text{O}\left(100\text{F}\times N^{2}\right), \end{array}$$

considering the worst-case performance scenario.

The evaluation of these two algorithms showed that, if *N* is bigger than 10 samples, Limada had worse performances than AdaBandFlt, regardless of the hardware. However, 10 samples are extremely few and not feasible for a reliable threshold identification (at the most commonly used frequency of 10 kHz, 10 samples mean 1 ms that is less than usual estimation of one AP duration).

### 3.3. Proposed Architecture and Performance Analysis

**Figure 7:**
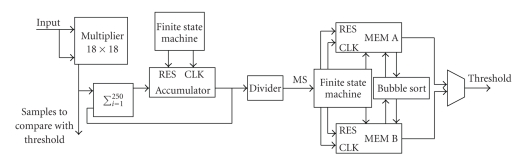
Architectural blocks to compute a threshold value.

To verify the feasibility of AdaBandFlt implementation on FPGA, CLBs number, memory occupation and timing were evaluated. Figure 7 shows the blocks architecture to compute the threshold value for 1 channel. The input is a 12 bit data stream, coming from an analog to digital converter (ADC). To compute the threshold, the system has to evaluate the RMS value of 250 samples (i.e., 10 ms windows, assuming a sample frequency equal to 25 kHz); after computing the RMS for a window, the system has to collect 100 RMS values and to sort them. The threshold is the 25th percentile of this ordered distribution. Note that we simplified the algorithm doing the following assumption.

- we used a square threshold values (MS) and compare it with square samples, avoiding to compute the root (8):
  (8) $$25\text{th}\;\text{percentile}\left(\text{MS}\right)={\left(25\text{th}\;\text{percentile}\left(\text{RMS}\right)\right)}^{2},$$
- we skipped out the problem of division using FPGA, rounding 250 samples to 256; this allowed us to calculate the RMS only shifting the point of 8 positions.

Thus, the input enters the embedded multiplier 18 × 18, its output is a 24 bit word (we decided to use one more bit to keep track of sign) and it is recursively added to other samples; to perform the sum, the adder utilizes the accumulator, intrinsically controlled by a finite state machine, shared with all channels. The division result is, in the worst case, a 32 bit word, 24 bits before the point and 8 bits after the point. To a first approximation, we decided to define the MS value only with the first 24 bits. In terms of hardware occupation, to compute the MS value we need

- 1 differential IOB,
- 1 embedded multiplexer,
- 32 LUTs for the adder [30] and 32 flip-flops for the accumulator, matching 4 CLBs,
- 1 32 bit shift register, that is, 2 CLBs,
- 1 CLB for the finite state machine.

After computing the MS value, it is memorized. Specifically, the first 100 MS values are memorized in MEM A and the second 100 RMS^2^ values are memorized in MEM B, while values contained in MEM A are sorted by the BubbleSort logic. The following step is dual: MEM A is filled while values in MEM B are sorted. This is controlled by a finite state machine, shared with all channels. The finite state machine also manages the computation of the 25th percentile index, counted as follows:

(9) $$I_{k}=\left(0.5+\frac{n\times k}{100}\right),$$

with *n* the sorted vector length and *k* the *k*th percentile. Thus, in the instance considered (*n * = 100, *k * = 25), *I_k_* is about 25: computing the 25th percentile means choosing the 25th sample in the sorted vector. Therefore, the first value of threshold comes out from MEM A, the second from MEM B and so on. In terms of hardware occupation, to compute the threshold value we need

- 1 CLB for the finite state machine,
- 2.3 kbits of RAM for each memory block (i.e., 4.6 kbits),
- 50 CLBs for BubbleSort (i.e., *N*/2 pipelined stages, with *N* the length of the sorted input vector) [31],
- 24 2 : 1 multiplexers.

**Figure 8:**
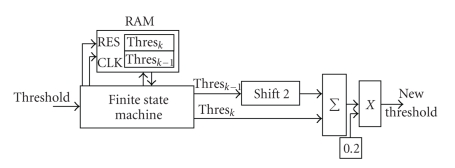
Hardware blocks to update the threshold.

After computing the 24 bit threshold value, the system puts it into a RAM 24 bits location, named Thres_k-1_ in Figure 8; likewise, it puts the following threshold value in Thres_k_. Then, the threshold values are updated as in (4): Thres_k-1_ is multiplied by 4 (i.e., shifted left of 2 positions), added to Thres_k_ and multiplied by 0.2. Finally, Thres_k_ becomes Thres_k-1_ and the new threshold is memorized in Thres_k _ location. All these operation are controlled by a finite state machine, shared with all channels.

In terms of hardware occupation, to update the threshold value we need

- 48 bits of RAM,
- 1 CLB for the finite state machine,
- 1 32 bit shift register, that is, 2 CLBs,
- 3 CLBs for the adder,
- 5 CLBs for the multiplier.

Pipelined with threshold values counting, the system detects spikes. First, it compares the *i*th squared sample with the threshold to find over-threshold values; then, it verifies if the detected value is a maximum (or a minimum), as follows:

(10) $$\left|x_{i}^{2}\right|>\left|\text{Thres}\right|\&\left|x_{i}^{2}\right|>\left|x_{i-1}^{2}\right|\&\left|x_{i}^{2}\right|>\left|x_{i+1}^{2}\right|.$$

We decided to realize these comparison subtracting each couple and looking at the sign. Thus (10) can be rewritten as follows:

(11) $$\begin{array}{cc} \text{PS}\left(\left|x_{i}^{2}\right|-\left|\text{Tresh}\right|\right) & \&\;\text{PS}\left(\left|x_{i}^{2}\right|-\left|x_{i-1}^{2}\right|\right)\\ & \&\;\text{PS}\left(\left|x_{i}^{2}\right|-\left|x_{i+1}^{2}\right|\right), \end{array}\;$$

with PS = positive signed.

This could be realized using 1 LUT for each couple of compared bits; to compose the comparison of complete words, additional XOR and AND gates are required [25]. Approximately, we need 3 CLBs for each word comparison (24 bit words), that is 9 CLBs for a detection.

With the design outlined above, we estimated FPGA occupation. Taking into account all 64 MEA channels, AdaBandFlt, mapped on FPGA, requires

- 64 differential IOB,
- 64 dedicated multipliers,
- 4803 CLBs,
- 291 kbits of RAM,
- 1536 dedicated 2:1 multiplexers.

Therefore, we identified two suitable solution for AdaBandFlt implementation. 

The first one envisages the use of a single FPGA; we identified the device XC3S5000 of the Xilinx Spartan-3 family, whose architecture satisfies our needs:

- 300 differential IOB,
- 104 dedicated multipliers,
- 8320 CLBs,
- 1872 kbits of block RAM,
- 65 k 2 : 1 dedicated multiplexers.

This gives a utilisation of around 58% of the FPGA, which seems a comfortable margin [30]. Furthermore, we analyzed RAM occupation in detail. The block RAM of XC3S5000 is ordered in 104 18 kbit blocks; the memory organization, suitable for our data, is 512×32 bit. We counted to use one block for each couple of RAM A and RAM B used to compute threshold values (one block for each channel), addressing RAM A from 1 to 100 and RAM B from 129 to 139 to simplify this task. This allow to sort RAM A and to fill RAM B almost contemporaneously. Moreover, even if XC3S5000 is the biggest device within Spartan-3 family, it is not too expensive (less than $120).

The second solution envisages the use of two smaller FPGAs (one every 32 channels), the XC3S4000 of the Xilinx Spartan-3 family, each containing

- 300 differential IOB,
- 96 dedicated multipliers,
- 6912 CLBs,
- 432 kbits of distributed RAM, organized in 96 18 kbit blocks,
- 54 k 2 : 1 dedicated multiplexers.

This gives a utilisation of around 35% of each FPGAs. Differently from the first solution, this design allows a modular approach, useful for future improvement. Furthermore, the device is rather cheap: in 2004, the volume pricing was under $100 for the XC3S4000 in 250 unit quantities [32].

Finally, we identified the BubbleSort as the bottleneck of our architecture and we decided to evaluate it in terms of temporal requirements, verifying if its timing was consistent with other operations. To sort a *n*-length vector, bubble sort makes *n* − 1 steps through the data. In each step, adjacent elements are compared and swapped if necessary. Notice that after the first pass through the data, the largest element in the sequence has bubbled up into the last array position. In general, after *k* passes through the data, the last *k*  elements of the array are correct and need not be considered any longer. Thus, we hypothesized 3 clock cycles for each comparison, *n*/2 clock cycles for each couple of values in the vector and *n* − 1 clock cycles for the whole sorting [33]. Since our device sorts a vector after collecting 100 windows of 250 samples each, there is a 500 ms delay between two sorting steps even considering the maximum sampling frequency (i.e., 50 kHz) of the data acquisition system. On the other hand, the device needs about 15 000 clock cycles to sort a 100 elements vector; even considering a slow FPGA inner frequency (i.e., 10 MHz), this means that it takes about 1.5 ms to sort a vector, that gives us a secure margin. Moreover, while *n*-1 passes through the data are required to guarantee that the list is sorted in the end, it is possible for the list to become sorted much earlier. When no exchanges at all are made in a given pass, then the array is sorted and no additional steps are required. A minor algorithmic modification would be to count the exchanges made in a pass, and to terminate the sort when none are made, decreasing the space occupation.

### 3.4. Spikes Classification on Simulated Signal

**Figure 9:**
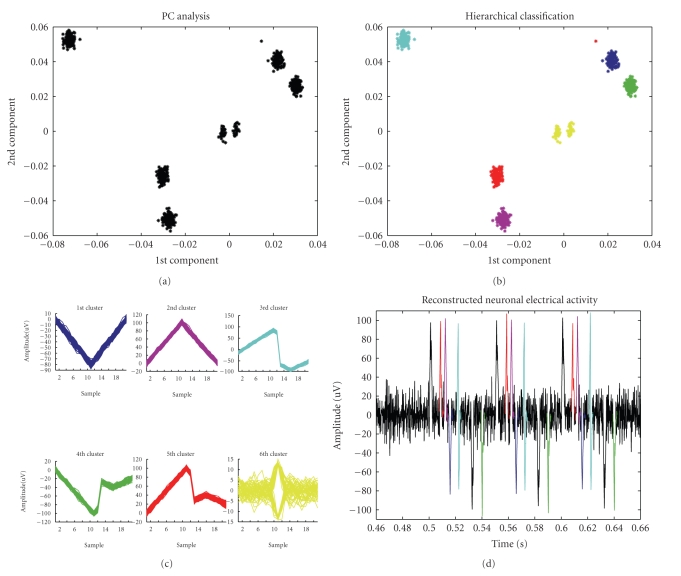
(a) Simulated data projected along the first two principal components. (b) Simulated data grouped and classified in the first two principal components space. (c) The 6 clusters identified by the hierarchical classification algorithm. (d) Reconstructed simulated signal (200 ms).

After AdaBandFlt was selected as the best noise level estimation method for our purposes, the whole spike detection algorithm was applied to the simulated neuronal signal described in Section 2.2; then, APs detected became the input for the classification algorithm. They were projected along the first two principal components (Figure 9(a)) to get APs into separated groups. It was already demonstrated [34] that 1st and 2nd component eigenvalues can represent the useful characteristics of classification. 

Then, hierarchical classifier yielded an accurate classification (Figure 9(b)). The defined maximum number of cluster was settled at 7: this value matches with a likely maximum number of cellular bodies in the electric field registered by each electrode plus one or two noise sources.

The algorithm identified 6 clusters plus one single element cluster (i.e., formed by only one element) that was rejected after the analysis. In Figure 9(c) all waveforms for each class are shown: 5 of them match exactly the 5 well-known classes of waveform artificially made up in the simulated neuronal signal. On the other hand, the yellow cluster is formed by two data groups (i.e., data projections around zero in Figures 9(a) and 9(b)) representing the cluster of false positive detected spike (i.e., peak-to-peak amplitude less than 30 *μ*V). 

Therefore, the classification is satisfactory; moreover, we had chosen a less specific spike detection algorithm not to lose spikes and, thanks to this classifier, we were able to reject FP, classified all together. 

The final goal was the signal reconstruction using the classified waveforms. In Figure 9(d) we can single out all kind of waveform previously made up in the simulated neuronal signal.

### 3.5. Spikes Classification on Neuronal Spikes

After testing the spike detection algorithm on 300 s real data, hierarchical classification was performed; classifier performances were evaluated by visual inspection on a portion of signal (5 s).

**Figure 10:**
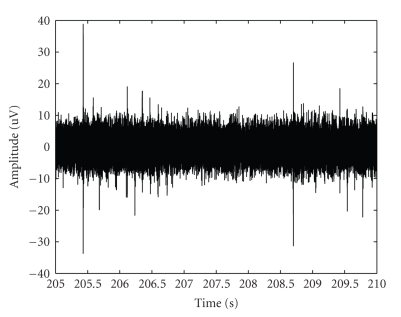
Neuronal electrical activity (5 s) recorded by MEA60 pre-amplifier.

**Figure 11:**
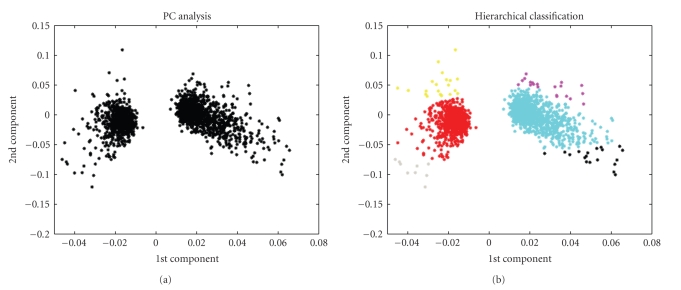
(a) Electrical activity projected along the first two principal components. (b) Hierarchical classifier output. Real data grouped and classified in the first two principal components space.

In Figure 10 an example of 5 s neuronal electrical activity recordings is presented. In Figure 11(a) waveforms projections on the 1st and the 2nd component eigenvalues are shown, then, the data are classified (Figure 11(b)).

**Figure 12:**
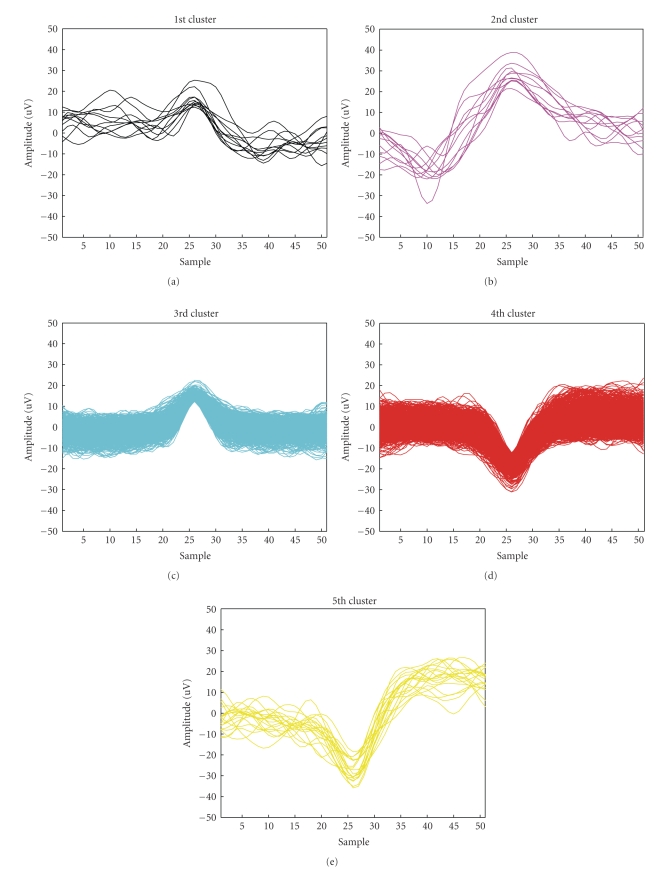
Identified clusters.

Figure 12 shows the clusters identified; each cluster amplitude and morphological characteristics are different. Note that the algorithm, also tested on real signal, distinguishes false positive detected spikes (i.e., Figures 12(c) and 12(d), clusters centred around zero in eigenvalues projection).

**Figure 13:**
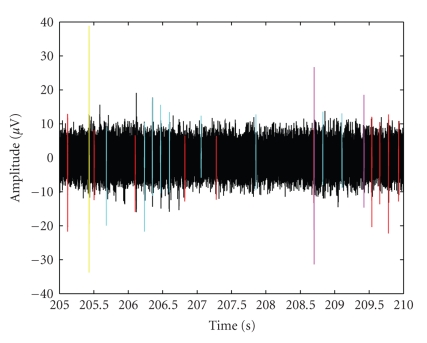
Reconstructed neuronal activity (5 s).

Finally, the neuronal electrical activity is reconstructed: in Figure 13 a 5 s segment is illustrated, for simplicity. It is possible to clearly distinguish red and light-blue spikes, that represent, respectively, positive and negative noise clusters, a yellow AP, biphasic but with no extreme polarization, few pink APs, still biphasic and prevalently positive raised, and some black peaks, essentially monophasic 40 *μ*V waveforms. 

## 4. Conclusions

In the context of extracellular electrophysiology, an efficient and reliable identification of spikes has to be reached. Furthermore, it's important to distinguish between application-specific and generalized methods [35]. In this paper we have proposed an application-specific algorithm, aimed at future hardware integration (on FPGA and DSP) for real-time long term acquisitions. 

The threshold-amplitude spikes detection method computes threshold as a multiple of basal noise level; 5 noise estimation methods were developed in Matlab (The Mathworks, Natick, USA) and their performances were statistically evaluated, both on simulated neuronal signal and on real electrical activity, recorded by MEA60 (Multi Channel Systems MCS, Gmbh). The characteristics we decided to favour were (a) the adaptability because, during long-term recordings, noise levels often drift on a time-scale of hours and (b) the minimum number of false negative and the sensibility, in order not to lose spikes because of the nonreversibility of the spike detection procedure. The statistical analysis identified two algorithms, AdaBandFlt and Limada, satisfying these requirements. Then, we evaluated their behaviours using the Big O Notation, that determined the minor complexity of AdaBandFlt, a quick adaptive noise estimation method, based on the evaluation of the 25th percentile of 1 s signal's RMS distribution. We decided to develop spike detection on FPGA to reach fast performances, and we assessed its hardware requirements, in terms of CLBs number, memory and timing request. We identified two feasible solution: the first one envisages the use of one FPGA (XC3S5000, Xilinx Spartan-3) that satisfied hardware requirements; the second one allows modular development utilising two smaller FPGAs, each for 32 channels (XC3S4000, Xilinx Spartan-3). 

Realizing the spike classification algorithm, we favoured a shape clustering and classification being automatic and reliable. After spike detection, the software extracts waveforms and sorts them around time stamps. Then, it bundles waveforms into groups with PCA and classifies APs with a hierarchical classifier, identifying false positive detected spikes, too. The classification algorithm was tested on simulated signal, comparing its output to the artificially designed waveforms; moreover, the classifier performances were evaluated on real data, comparing visual inspection results to the morphology of the identified clusters. The analysis stated the goodness of clustering procedure. We decided to develop PCA and hierarchical classifier on DSP, as already done in literature [27–29].

Therefore, the developed spike detection and classification algorithm has the highest performances in order to achieve long period recordings of neuronal cultures' electrical activity, avoiding data leakage and allowing its future hardware implementation.

Having verified its feasibility, in future works we'd like to develop an integrated hardware, composed of an high-speed device (FPGA) in order to guarantee a real-time detection of spikes and to save only their templates for the following classification, integrated in a DSP. Using high speed hardware devices, AdaBandFlt and the PCA-hierarchical classifier will be able to execute on-line detection and real time waveforms analysis, reducing data storage problems.

The system has many applications, from high-throughput pharmacological screening, to neuro pharmacology and monitoring effects of drugs and toxins (neurotoxicity), to neuroplasticity researches.

## Figures and Tables

**Algorithm 1:**
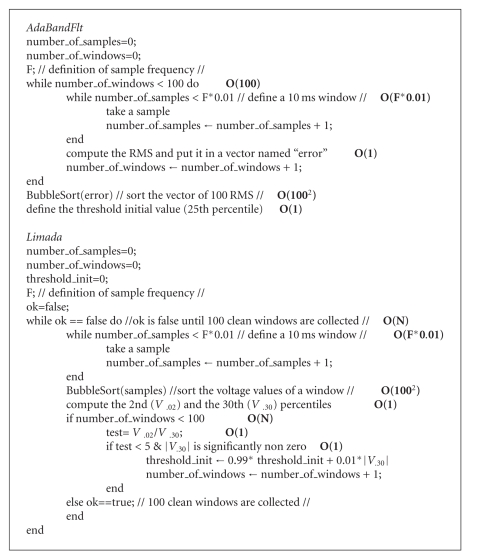
Pseudocode for AdaBandFlt and Limada.
